# A Range-Independent Disparity-Based Calibration Model for Structured Light Pattern-Based RGBD Sensor

**DOI:** 10.3390/s20030639

**Published:** 2020-01-23

**Authors:** Wenbin Li, Yaxin Li, Walid Darwish, Shengjun Tang, Yuling Hu, Wu Chen

**Affiliations:** 1 Shenzhen Research Institute, The Hong Kong Polytechnic University, Shenzhen 518057, China; wu.chen@polyu.edu.hk; 2 Department of Land Surveying and Geo-Informatics, The Hong Kong Polytechnic University, Hong Kong, China; yaxin.pu.li@connect.polyu.hk (Y.L.); lindsay444@outlook.com (Y.H.); 3 Geomatics Engineering Lab, Civil Engineering Department, Faculty of Engineering, Cairo University, Cairo 12613, Egypt; dawalid@etrovub.be; 4 Department of Electronic and Informatics, Faculty of Engineering, Vrije Universiteit Brussel, 1050 Brussels, Belgium; 5 Guangdong Key Laboratory of Urban Informatics & Shenzhen Key Laboratory of Spatial Smart Sensing and Services & Guangdong Laboratory of Artificial Intelligence and Digital Economy (SZ) & Research Institute for Smart Cities, School of Architecture and Urban Planning, Shenzhen University, Shenzhen 518050, China; shengjuntang@szu.edu.cn

**Keywords:** RGBD sensor, calibration model, disparity

## Abstract

Consumer-grade RGBD sensors that provide both colour and depth information have many potential applications, such as robotics control, localization, and mapping, due to their low cost and simple operation. However, the depth measurement provided by consumer-grade RGBD sensors is still inadequate for many high-precision applications, such as rich 3D reconstruction, accurate object recognition and precise localization, due to the fact that the systematic errors of RGB sensors increase exponentially with the ranging distance. Most existing calibration models for depth measurement must be carried out with different distances. In this paper, we reveal the mechanism of how an infrared (IR) camera and IR projector contribute to the overall non-centrosymmetric distortion of a structured light pattern-based RGBD sensor. Then, a new two-step calibration method for RGBD sensors based on the disparity measurement is proposed, which is range-independent and has full frame coverage. Three independent calibration models are used for the calibration for the three main components of the RGBD sensor errors: the infrared camera distortion, the infrared projection distortion, and the infrared cone-caused bias. Experiments show the proposed calibration method can provide precise calibration results in full-range and full-frame coverage of depth measurement. The offset in the edge area of long-range depth (8 m) is reduced from 86 cm to 30 cm, and the relative error is reduced from 11% to 3% of the range distance. Overall, at far range the proposed calibration method can improve the depth accuracy by 70% in the central region of depth frame and 65% in the edge region.

## 1. Introduction

The consumer-grade RGBD sensor has the potential to be used in many fields, such as 3D reconstruction [1,2], camera simultaneous localization and mapping (SLAM) [3], robotics exploration [4], and obstacle avoidance [5], due to its low cost and ability to provide depth with pixel-corresponded RGB information. Based on the ranging principle, consumer-grade RGBD sensors can be categorized into three groups: time-of-flight (ToF)-based RGBD sensors (i.e., Kinect V2 [6]), structured light pattern (SLP)-based RGBD sensors (i.e., Kinect V1 [6], Structure Sensor [7], Asus Xtion [8], Intel RealSense [9]), and stereo vision-based RGBD sensors (i.e., ZED camera [10]). Although all RGBD sensors provide the same data format, SLP RGBD has become one of the most popular RGBD solutions due to its low requirement for computational power [11]. Despite the wide use and popularity, the SLP-based RGBD sensor has one significant drawback: the errors in depth frame exponentially increase with ranging distance [6]. Existing applications of consumer-grade SLP RGBD sensor are mainly for gaming purposes, not for those requiring high-precision, such as SLAM and visual odometery [12]. With high-precision applications, the accuracy of depth measurement is crucial as errors in depth will be accumulated during the frame matching process, which will significantly affect the quality of final 3D point cloud and position products and may cause a frequent loss tracking problem. Thus, proper calibration of depth measurements is required for SLP RGBD sensors [13].

**Figure 1:**
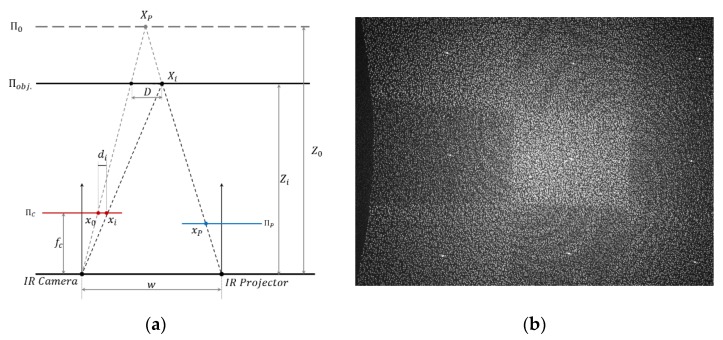
(**a**) Projection model of structured light depth sensor; (**b**) example of pre-structured pattern [14].

The SLP RGBD sensor calculates ranging information based on the disparity $d_{i}$ (pixel difference, $d_{i}=\left|x_{i}-x_{0}\right|$) between the reference pixel $x_{0}$ location of the pre-structured pattern $X_{p}$ in the reference plane $\Pi_{0}$ and the measure pixel location $x_{i}$ of captured pattern $X_{i}$ on the object plane $\Pi_{obj.}$ in the received image frame, as shown in Figure 1a. The reference pixel $x_{0}$ of the pre-structured pattern is fixed in the image plane $\Pi_{C}$. The pre-structured pattern as shown in Figure 1b, is a fixed pattern of light and dark speckles. The depth is calculated by triangulation against the pre-structured pattern. If the captured feature $x_{i}$ can be matched to the reference pixel $x_{0}$, the disparity $d_{i}$ can be obtained. For a commercial SLP RGBD sensor, information including the actual pattern $x_{p}$ in the projector plane $\Pi_{P}$ and the distance $Z_{0}$ from the IR projector to the reference plane $\Pi_{0}$ remains undisclosed.

For the object $X_{i}$, the depth $Z_{i}$ can be expressed as:

(1) $$Z_{i}=\frac{1}{\frac{1}{Z_{0}}+\frac{1}{f_{c}w}d_{i}}$$

where,

$Z_{0}$ is the reference distance from the sensor to the reference plane $\Pi_{0}$;
$f_{C}$ is the focal length of IR camera;
w is the baseline between the IR camera and IR projector;
$d_{i}$ is the disparity for $x_{i}$.

For commercial RGBD sensors, such as Structure Sensor and Kinect V1, the actual disparity $d_{i}$ in pixel unit is not available to the users. Instead, a normalized disparity $d_{i}^{\prime }$ value from 0 to 2047 is outputted. Normalized disparity is expressed as Equation (2), *m, n* are the normalized parameters that remain unknown:

(2) $$d_{i}^{\prime }=m\cdot d_{i}+n$$

Combine (1) and (2):

(3) $$Z_{i}=\frac{1}{\frac{1}{Z_{o}}-\frac{n}{f_{c}wm}+\frac{1}{f_{c}wm}d_{i}^{\prime }}=\frac{1}{\alpha +\beta d_{i}^{\prime }}$$

where: $\alpha =\frac{1}{Z_{o}}-\frac{n}{f_{c}wm},\;\beta =\frac{1}{f_{c}wm}$, α & β are fixed parameters of a RGBD sensor, and are provided by the manufacturer to calculate the depth $Z_{i}$ from the normalized disparity $d_{i}^{\prime }$. 

Early works on SLP RGBD calibration [6,15] described the systematic error in the depth frame as a function of radial distortion parameters and adopted the classic RGB camera distortion model [16] to correct the radially symmetric error on the SLP-based RGBD sensor. However, they failed to address the fact that the distortion increases with distance. Zhang and Zhang [17] proposed a depth dependent calibration model that separately fits a linear equation of the actual depth and the rotation angle for each pixel. The limitations for this method are the instability of the algorithm, the requirement of accurate initial parameters to avoid divergence, and the high computational cost. Similarly, Canessa et al. [18] proposed a labour intense empirical calibration model by using the sample images captured from 0.6 to 2 m to fit a second degree polynomial model for each pixel. Recently, Darwish et al. [19] proposed a calibration model, treating the depth distortion of a RGBD sensor as a combined centrosymmetric error of the IR camera distortion and IR projector distortion with same optical centre. A look up table of calibration parameters was calculated at different distance, i.e., from 0.5 to 3 m with 0.5 m interval. With insufficient investigation of the mechanism of how the IR camera and IR projector contribute to the overall non-centrosymmetric distortion of the RGBD sensor, and also being a distance-based calibration model, this method suffers large offsets in the edge area of depth frame and requires a look up table for parameters in different distance. Similar problems can be found in [20]. 

A visual camera or a RGB camera often have a centrosymmetric or near centrosymmetric distortion pattern. When the distortion model is adopted in RGBD sensor calibration, the assumption that the distortion of SLP RGBD is also centrosymmetric and can be modeled by a classic distortion model is accepted by many researchers [15,20,21,22]. However, compared to visual distortion that can use one model to represent the distortion, current calibration models for SLP RGBD sensors are much more complex due to the additional work such as fitting the model separately based on distance, the requirement of good initial parameters, and the larger number of parameters to provide full range coverage. The complexity of current calibration models as discussed later in this paper is mainly due to the negligence of the fundamental difference between the visual camera and the SLP RGBD sensor. One significant difference between visual camera and SLP RGBD sensor is that the distortion in the depth frame of the RGBD sensor is caused by both camera lens and projector lens while image distortion is only caused by the camera lens. In fact, due to the combined effects of camera and projector, the depth distortion is non-centrosymmetric rather than the centrosymmetric distortion in the visual camera. Therefore, the existing methods that only use a centrosymmetric distortion model to calibrate the depth distortion, for example the method of Darwish et al. [19], will inevitably suffer large offset in the frame edge area. The edge area is extremely sensitive to the high order part in the visual camera distortion model [16]. Furthermore, the target measurement of SLP RGBD sensors to apply the distortion model should be disparity rather than depth, as disparity is the raw measurements of distance measurement. A camera distortion model was originally designed for calibrating the distorted pixel caused by camera lens. Although a similar distortion pattern can be discovered in depth frames, it is actually a reciprocal product of the pixel difference (disparity) between the captured pattern and reference pattern. By analyzing the distortions of the camera and the projector separately, this paper proposes a new distortion model to calibrate disparity which can be applied to full range depth frames of SLP-based RGBD sensors. Compared to the current methods, the proposed method is distance independent.

**Figure 2:**
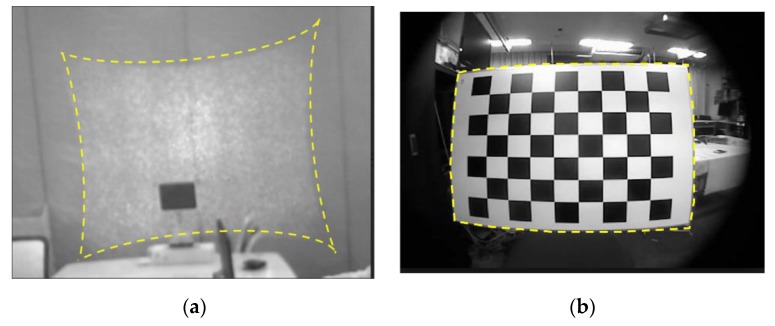
(**a**) Pincushion distortion of infrared (IR) projector; (**b**) barrel distortion of IR camera.

This paper firstly makes a comprehensive discussion of the sources and forms a mechanism of depth distortion of the SLP RGBD sensors. By compositing the calibration model that is strictly based on actual physical model of camera distortion and projector distortion, we propose a new method which can effectively and accurately calibrate the full range depth data. The proposed method calibrates the whole range depth with just one model and one set of parameters. Compared to the existing model that requires to separately fit calibration parameters for different distance range or a look up table of parameters, the proposed method largely improved the efficiency of the calibration procedure. By applying the proposed calibration model, we can accurately improve the ranging accuracy by 70% in the central area and 65% in the edge area of the depth frame. Two distortions and one infrared cone related bias will be used to model the overall systematic error of SLP sensors. The two distortions are a pincushion distortion caused by the projector lens and a barrel distortion caused by infrared camera lens, shown in Figure 2. A bias caused by the infrared cone that cannot illuminate the homogeneously for a unified pattern recognition performance [6,23] is also modeled in the proposed calibration method. As the systematic error is extremely sensitive to how these two distortions and the bias overlapped, accurately determining the forming mechanism of the overall distortion is crucial for calibration.

Then a two-step calibration procedure is proposed to overcome the problem that the commuter-grade RGBD sensor often remains a black box to the users. SLP RGBD sensors such as Kinect V1 [24] and Structure Sensor [7] only provide a normalized disparity unit, but the raw disparity measurement and the normalization parameters for disparity remain unknown. Other information, such as original reference pattern and the actual measurement of the captured pattern, is also unavailable to users. Such black box environment is extremely problematic for the sensor calibration. Although the camera distortion and camera internal parameters can be calibrated by traditional checkboard method [25], the projector distortion cannot be easily calibrated with existing methods [26,27,28] which all require knowing the original reference pattern and take measurement of the casted pattern in real world. The two-step calibration procedure is proposed to achieve a precise calibration procedure in such black box environment for RGBD sensor. The two-step calibration procedure includes: (1) the IR camera related distortion is calibrated with classic checkboard method; (2) the projector distortion in normalized disparity is modeled by a new combined objective function. 

The calibration method is evaluated by two experiments in an indoor environment. One is an experiment designed to test the performance of the calibration model. A set of test data with ground truth error is acquired in three different scenes: (1) flat surface, (2) non-flat surface, and (3) flat surface with distance variation. The ground truth error is obtained by plane fitting and compared with the modeled error generated from the proposed method. Comparison results demonstrate that the proposed method can precisely model the systematic error caused by non-centrosymmetric distortion of the RGBD sensor. The other one is an evaluation of proposed method in actual point cloud collected in the indoor environment. By comparing the calibrated and uncalibrated point cloud in the whole working range of the tested SLP sensor, the improvement of the calibration model can be demonstrated clearly. 

## 2. RGBD Distortion Model

To calibrate the SLP based RGBD sensor, it is important to understand how the error is introduced in the RGBD sensor and how that error affects the depth measurements. The radical systematic error appeared in the depth frame is mainly a combined result of the distortions caused by camera lens and projector lens. The infrared cone also causes a certain radical error to the depth frame. In this section, the mechanism of the non-centrosymmetric distortion of RGBD sensor will be revealed. 

The distortion appearing in the disparity measurement of SLP RGBD sensor can be traced back to the two optical lenses in its hardware, the projector lens and camera lens. The projector lens causes a pincushion distortion as shown in Figure 2a; and the camera lens causes a barrel distortion as shown in Figure 2b. As the pattern is casted in IR and can’t be captured by normal digital camera, Figure 2a is captured by a calibrated IR camera called MIQUS M3 [29]. Figure 2b is captured by the IR camera. Both pincushion and barrel distortion are optical distortions, therefore, they occur as a result of optical design and lens error. They both appear and overlap in the disparity measurement of a SLP RGBD sensor.

The data accessed by users are the normalized disparity unit, which is a normalized pixel difference between the pre-stored reference pattern and the captured pattern. The most basic observation of SLP RGBD sensor is the disparity $d_{i}$ between the pre-stored distortion-free reference pixel $x_{o}$ and the captured distortion affected measure pixel $x_{i}$. The distortion first appeared in the measured pixel $x_{i}$ and then passed down to the disparity and normalized disparity. 

The pincushion distortion occurred when the pre-stored pattern was casting through the projector lens into the object scene. As shown in Figure 2a, the reference pattern (rectangle shape) would be distorted into a pincushion shape (marked as yellow in Figure 2a). When the pincushion distortion pattern in the object space was then captured by the IR camera’s complementary metal–oxide–semiconductor (CMOS) sensor [30], the camera lens would cause additional barrel distortion (Figure 2b) to the already distorted casted pattern. As a result, the captured patterns, which is used to calculate disparity, contains the distortion caused by both IR projector lens and IR camera lens and two types of distortion overlapped in the image plane. Since the disparity, or normalized disparity, are the linear products of the captured pattern, the distortion model that is applicable to capture pattern in pixel unit can also be applied in the disparity or normalized disparity. 

Considering both camera and projector distortions, the measured disparity in pixel can be described as:

(4) $$d_{M}=d_{i}+\delta_{c}^{ep}+\delta_{p}^{ep}$$

where,

$d_{M}$ is the measurement value of disparity in pixel;
$d_{i}$ is the true value of disparity in pixel with no error;
$\delta_{c}^{ep}$ is the distortion error caused by camera lens in pixel;
$\delta_{p}^{ep}$ is the distortion error caused by projector lens in pixel.

According to Zhang [17], the optical distortion of the optical lens can be modeled as:

(5) $$x_{u}=x_{d}+\left(x_{d}-x_{c}\right)\left(K_{1}r^{2}+K_{2}r^{4}+\cdots \right)+\left(P_{1}\left(r^{2}+2{\left(x_{d}-x_{c}\right)}^{2}\right)+2P_{2}\left(x_{d}-x_{c}\right)\left(y_{d}-y_{c}\right)\right)\;y_{u}=y_{d}+\left(y_{d}-y_{c}\right)\left(K_{1}r^{2}+K_{2}r^{4}+\cdots \right)+\left(2P_{1}\left(x_{d}-x_{c}\right)\left(y_{d}-y_{c}\right)+P_{2}\left(r^{2}+2{\left(y_{d}-y_{c}\right)}^{2}\right)\right)$$

where,

$\left(x_{d},y_{d}\right)$ = distorted image point as projected on image plane;
$\left(x_{u},y_{u}\right)$ = undistorted image point as projected by an ideal pinhole camera;
$\left(x_{c},y_{c}\right)$ = distortion centre;
$K_{n}=n^{th}$ radial distortion coefficient;
$P_{n}=n^{th}$ tangential distortion coefficient; and
$r=\sqrt{{\left(x_{d}-x_{c}\right)}^{2}+{\left(y_{d}-y_{c}\right)}^{2}}$.

Camera distortion is normally barrel distortion with a negative term for $K_{1}$, and projector distortion is often pincushion distortion with a positive $K_{1}$. 

**Figure 3:**
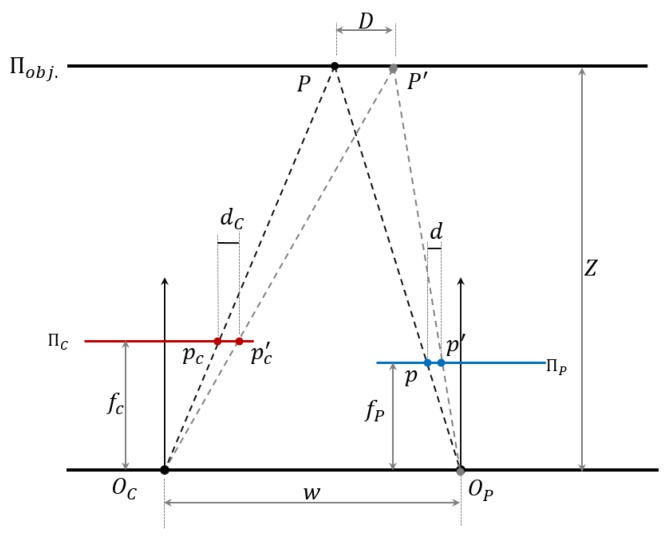
Relationship between the project distortion in object space and in image plane.

Both the camera-caused barrel distortion and projector-caused pincushion distortion are uncorrelated to the distance. The camera-caused barrel distortion in the disparity is a fixed centrosymmetric distortion with a centre at the optical centre of the IR image plane for the whole distance. For the pincushion distortion of the projector lens, it can be proved that the casted projector distortion’s effect in the image plane is uncorrelated with distance, and same as the barrel distortion caused by the camera lens, the pincushion distortion on the image plane should be a fixed distortion for all distances. It is because the ratio between the original pincushion distortion in projector plane and the captured pincushion distortion in image plane is a constant value for all the distances. Figure 3 illustrates that the pixel distortion d in projector plane $\Pi_{P}$ caused by the projector lens in the object scene has a fixed relationship with the pincushion distortion $d_{C}$ that appeared in the image plane $\Pi_{C}$.

Where,

$\left(f_{P}\;,f_{C}\right)$ is the focal length of projector lens and camera lens;
$\left(O_{P},\;O_{C}\right)$ is the optical centre of the projector lens and camera lens;
$\Pi_{obj}$ is the object plane;
$\Pi_{P}$ is the projector pattern plane;
$\Pi_{C}$ is the camera image plane;
$\left(p^{\prime },\;p\right)$ is the undistorted and distorted pattern location in projector pattern plane $\Pi_{P}$;
$\left(P^{\prime },P\right)$ is the undistorted and distorted pattern location casted into the object plane $\Pi_{obj}.$;
$\left(p_{C}^{\prime },\;p_{C}\right)$ is the undistorted and distorted pixel captured by camera in image plane $\Pi_{C}$;
d is the pixel difference in projector plane $\Pi_{P}$;
$d_{C}$ is the pixel difference in image plane $\Pi_{C}$;
D is the location difference in object plane $\Pi_{obj.}$;
w is the baseline between camera centre and projector centre;
Z is the distance from baseline to object plane.

As shown in Figure 3, pixel difference d is the pincushion distortion in the projector plane $\Pi_{P}$. When this distortion is projected into the object plane $\Pi_{obj}$, it is presented as the location difference D between the actual casting location P and the undistorted location $P^{\prime }$. As here we are discussing the projector distortion’s effect on the image plane, the camera distortion is not considered for better visualization. In actual sensor measurement, the projector distortion and camera distortion are overlapped in image plane and have a combined effect on the distorted pixel $p_{C}$. With no camera lens barrel distortion considered, the location difference D in the object plane $\Pi_{obj}.$ will be captured as the pixel difference $d_{C}$ in image plane $\Pi_{C}$. From Figure 3, the following equation can be obtained:

(6) $$\{\begin{array}{l} \frac{d}{D}=\frac{f_{P}}{Z}\\ \frac{d_{c}}{D}=\frac{f_{c}}{Z} \end{array}$$

Combine the two parts in Equation (6), the following relationship can be obtained:

(7) $$d_{c}=\frac{f_{c}}{f_{P}}d$$

Since $\frac{f_{C}}{f_{p}}$ is a constant value, therefore, no matter what the distance Z is, the pixel shift $d_{C}$ in the image plane $\Pi_{C}$ caused by the projector lens follows the same model as the original pincushion distortion in the projector plane $\Pi_{P}$ and just with an additional scale $\frac{f_{C}}{f_{p}}$. The scale $\frac{f_{C}}{f_{p}}$ can be put into the parameters $K_{1}\;K_{2}\;P_{1}\;P_{2}$ of Equation (5) as new parameters $K_{1}^{\prime }\;K_{2}^{\prime }\;P_{1}^{\prime }\;P_{2}^{\prime }$ in the distortion model. Therefore, the distortion impact from projector in image plane $\Pi_{C}$ follows the same pincushion model in projector plane $\Pi_{P}$ and is not correlated to the distance from the projector to the object. 

**Figure 4:**
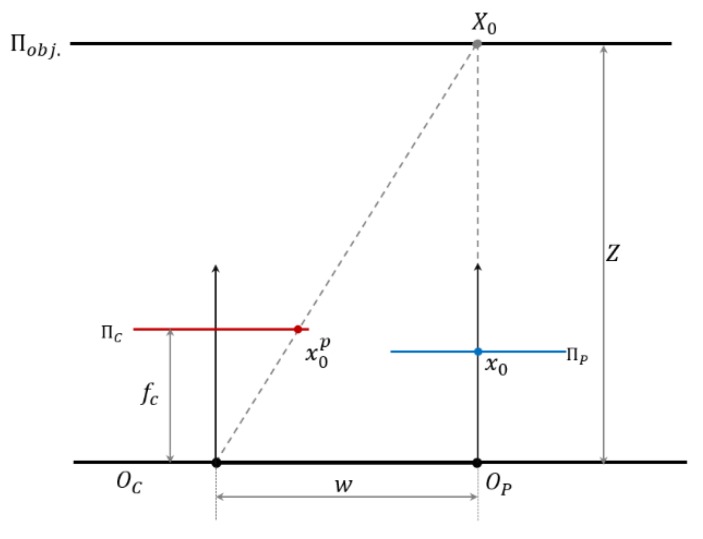
Projector centre in image plane.

Both camera barrel distortion and projector pincushion distortion are centrosymmetric distortion in the normalized disparity unit. The camera barrel distortion is constant at the optical centre of the IR image plane for all the distance range. For the pincushion distortion appeared in the image plane, although it is still a centrosymmetric distortion, it has varying distortion centre $\left(x_{0}^{p},y_{0}^{p}\right)$ based on the distance Z. In the SLP RGBD sensor’s chipset, the disparity is only calculated from the pixel difference in the x axis, and for this reason, the y coordinates $y_{0}^{p}$ of the pincushion distortion centre are always equal to 0. The x coordinates of $\left(x_{0}^{p},y_{0}^{p}\right)$ varies based on the distance. Based on the geometry shown in Figure 4, the image coordinates of projector centre $\left(x_{0}^{p},y_{0}^{p}\right)$ in image plane can be calculated as Equation (8).

(8) $$\{\begin{array}{l} x_{0}^{p}=\frac{f_{c}w}{Z}\\ y_{0}^{p}=0 \end{array}$$

**Figure 5:**
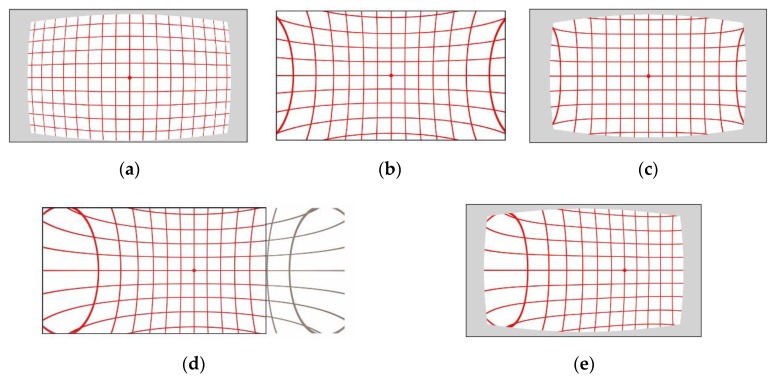
Different overlapping distortion in image plane. (**a**) Centrosymmetric barrel distortion with centre in the optical centre of image frame; (**b**) centrosymmetric pincushion distortion with centre in the optical centre of image frame; (**c**) centrosymmetric overlapped distortion with centre in the optical centre of image frame; (**d**) centrosymmetric pincushion distortion with centre shifted to the side of image frame; (**e**) non-centrosymmetric overlapped distortion with barrel distortion centre in the optical centre of image frame and pincushion distortion centre in the shifted side of image frame.

Although both the barrel distortion and pincushion are centrosymmetric distortion in the disparity unit, the combined overall distortion is a non-centrosymmetric distortion due to the pincushion distortion’s varying centre. If the centrosymmetric barrel distortion and pincushion distortion have the same distortion centre in the image plane, as shown in Figure 5a,b, then the overlapped distortion, shown in Figure 5c, is also a centrosymmetric distortion with the distortion in the optical centre of the image frame. According to Equation (8), the pincushion distortion centre $\left(x_{0}^{p},y_{0}^{p}\right)$ in the image frame is always different from the barrel distortion centre. Although the pincushion distortion in a shifted centre is still a centrosymmetric pattern (Figure 5d), its overlapping result with a barrel distortion of different distortion centre will become a non-centrosymmetric pattern, as shown in Figure 5e. Therefore, the overall distortion in the SLP RGBD sensor has a non-centrosymmetric pattern. The typical distortion pattern is similar to that illustrated in Figure 5e, with distortion centre (the least distorted part) shifted from the optical centre to the side of the projector module and the far side away from the projector centre suffering more severe distortion than the other side. 

Normalize the disparity in Equation (4) using Equation (2):

(9) $$d_{M}^{\prime }=m\left(d_{i}+\delta_{c}^{ep}+\delta_{p}^{ep}\right)+n=d_{i}^{\prime }+m\delta_{c}^{ep}+m\delta_{p}^{ep}$$

where,

$d_{M}^{\prime }$ is the measurement value of normalized disparity;
m, n are the normalize parameter of disparity;
$d_{i}$ is the true value of disparity in pixel with no error;
$\delta_{c}^{ep}$ is the distortion error caused by camera lens in pixel;
$\delta_{p}^{ep}$ is the distortion error caused by projector lens in pixel;
$d_{i}^{\prime }$ is the true value of normalized disparity.

Based on the Brown’s model [31], $\delta_{c}^{ep}$ and $\delta_{p}^{ep}$ can be expanded to radial distortion part and tangential distortion part as:

(10) $$\{\begin{array}{l} \delta_{c}^{ep}=\delta_{c}^{rad.}+\delta_{c}^{tang.}\\ \delta_{p}^{ep}=\delta_{p}^{rad.}+\delta_{p}^{tang.} \end{array}$$

The distortion model can be expressed as:

(11) $$\{\begin{array}{l} \delta^{rad.}=x_{i}\left(K_{1}r^{2}+K_{2}r^{4}+K_{3}r^{6}\right)\\ \delta^{tang.}=P_{1}\left(r^{2}+2x_{i}{}^{2}\right)+P_{2}x_{i}y_{i} \end{array}$$

where,

$\delta_{c}^{rad.}$ is the radial distortion of the IR camera;
$\delta_{c}^{tang.}$ is the tangential distortion of the IR camera;
$\delta_{p}^{rad.}$ is the radial distortion of the IR projector;
$\delta_{p}^{tang.}$ is the tangential distortion of vIR projector;
$r=\sqrt{x_{i}^{2}+y_{i}^{2}}\;x_{i}=x_{i}^{m}-x_{0}$, $y_{i}=y_{i}^{m}-y_{0}$;
$\left(x_{0},y_{0}\right)$ is the camera or projector centre;
$\left(x_{i}^{m},y_{i}^{m}\right)$ is measured image coordinate;
$K_{1},K_{2},K_{3}$ is the parameters of radial distortion model;
$P_{1},P_{2}$ is the parameters of tangential distortion model.

In this way, Equation (10) can be further expanded as:

(12) $$\left\{ \begin{array}{l} \delta_{c}^{ep}=x_{i}^{c}\left(K_{1}^{c}r_{c}^{2}+K_{2}^{c}r_{c}^{4}+K_{3}^{c}r_{c}^{6}\right)+P_{1}^{c}\left(r_{c}^{2}+2{x_{i}^{c}}^{2}\right)+P_{2}^{c}x_{i}^{c}y_{i}^{c}\\ \delta_{p}^{ep}=x_{i}^{p}\left(K_{1}^{p}r_{p}^{2}+K_{2}^{p}r_{p}^{4}+K_{3}^{c}r_{p}^{6}\right)+P_{1}^{p}\left(r_{p}^{2}+2{x_{i}^{p}}^{2}\right)+P_{2}^{p}x_{i}^{p}y_{i}^{p} \end{array}\right.$$

where,

$K_{1}^{c},\;K_{2}^{C},\;K_{3}^{C},\;P_{1}^{C},\;P_{2}^{C}$ are the distortion parameters of the IR camera;
$r_{c}=\sqrt{{x_{i}^{c}}^{2}+{y_{i}^{c}}^{2}}$; $x_{i}^{c}=x_{c}^{m}-x_{0}^{c}$; $y_{i}^{c}=y_{c}^{m}-y_{0}^{c}$;
$\left(x_{0}^{c},y_{0}^{c}\right)$ is the centre point of camera;
$\left(x_{c}^{m},y_{c}^{m}\right)$ is the measured image position;
$K_{1}^{P},\;K_{2}^{P},\;K_{3}^{P},\;P_{1}^{P},\;P_{2}^{P}$ are the distortion parameters of the IR projector;
$r_{p}=\sqrt{{x_{i}^{P}}^{2}+{y_{i}^{p}}^{2}}$; $x_{i}^{p}=x_{p}^{m}-x_{0}^{p}$; $y_{i}^{p}=y_{p}^{m}-y_{0}^{p}$;
$\left(x_{0}^{p},y_{0}^{p}\right)$ is the centre point of projector distortion model in image coordinates;
$\left(x_{p}^{m},y_{p}^{m}\right)$ is the measured image position without the influence of camera distortion.

(13) $$\{\begin{array}{l} x_{p}^{m}=x_{c}^{m}+\delta_{c}^{ep}\\ y_{p}^{m}=y_{c}^{m}+\delta_{c}^{ep} \end{array}$$

Combine (9), (10) & (12), the proposed calibration model in normalized disparity can be expressed as:

(14) $$\begin{array}{ll} \delta_{d_{i}}=d_{M}^{\prime }-d_{i}^{\prime }= & mx_{i}^{c}\left(K_{1}^{c}r_{c}^{2}+K_{2}^{c}r_{c}^{4}+K_{3}^{c}r_{c}^{6}\right)+mP_{1}^{c}\left(r_{c}^{2}+2x_{i}^{c}{}^{2}\right)+mP_{2}^{c}x_{i}^{c}y_{i}^{c}\\ & \;+mx_{i}^{p}\left(K_{1}^{p}r_{p}^{2}+K_{2}^{p}r_{p}^{4}+K_{3}^{c}r_{p}^{6}\right)+mP_{1}^{p}\left(r_{p}^{2}+2{x_{i}^{p}}^{2}\right)+mP_{2}^{p}x_{i}^{p}y_{i}^{p} \end{array}$$

Therefore, for the SLP-based RGBD sensor, the distortion error in disparity consists of two parts, one is a barrel distortion caused by camera lens with fixed location at optical centre. The other is pincushion distortion caused by the projector lens with a varying location over the camera image plane. In practice, since the actual distance Z of projector to the object scene is often unknown, the proposed calibration model uses the measured distance $Z_{M}$ in Equation (8) to calculate the distortion centre $\left(x_{0}^{p},y_{0}^{p}\right)$ as an alternative. 

## 3. Calibration of Structured Light Pattern (SLP) RGBD Sensor

In the SLP sensor, both camera distortion and projector distortion occur. In the image plane, the camera distortion is fixed as its optical centre, and the projector lens distortion has a varying optical centre in the image plane according to the distance from projector to the object. Therefore, if the distortion model for the camera and projectors is known, the combined distortion can be calculated proximately by the measured distance. The distortion calibration for infrared camera can be conducted in the traditional ways. But for the raw observation, the structured pattern of infrared projector of RGBD sensor is inaccessible, and there is no way to determine the projector distortion model by using the existing method [32] that requires a known reference pattern and ability to take measurement of the actual distorted casted pattern in the object space. The RGBD sensor only provides a normalized disparity unit that contains the combined distortion. To calibrate such combined non-centrosymmetric distortion, a two-step calibration procedure is proposed. 

**Figure 6:**
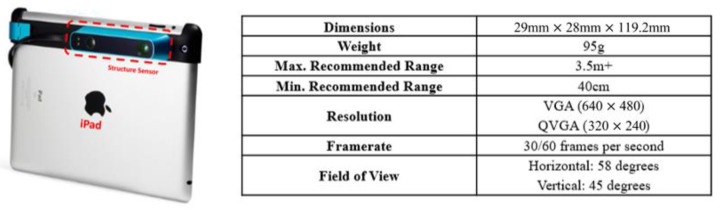
Structure sensor specifications.

A two-step calibration procedure is designed to specifically calibrate the SLP-based RGBD sensors. The RGBD sensor used in this paper is the Structure Sensor from Occipital [7], shown in Figure 6. It is a SLP-based RGBD sensor released in 2013. Structure Sensor shares the same ranging principle with Kinect V1 and Asus Xtion that are all based on the PrimeSense technology for SLP ranging [11]. The calibration procedure developed in this paper is also applicable for other PrimeSense-based SLP RGBD sensor. To obtain the parameters of the proposed calibration model (Equation (14)), the standard checkboard calibration method for the RGB camera is first used to get the parameters related to the infrared camera lens. Direct sunlight or infrared light is used to illuminate the checkboard. Then a test field is set up to calibrate the distortion related to the infrared projector. Sample data is captured at a different distance and plane fitted to estimate the distortion parameters. The camera distortion and projector distortion are combined based on the varying focal centre relating to the measured distance. At a different distance, there will be different combined effects on the depth frame due to the shifting location of the projector focal centre on the image plane. The remaining error after modelling the camera distortion and projector distortion is regarded as bias caused by the infrared cone. 

### 3.1. Data Acquisition and Data Processing

**Figure 7:**
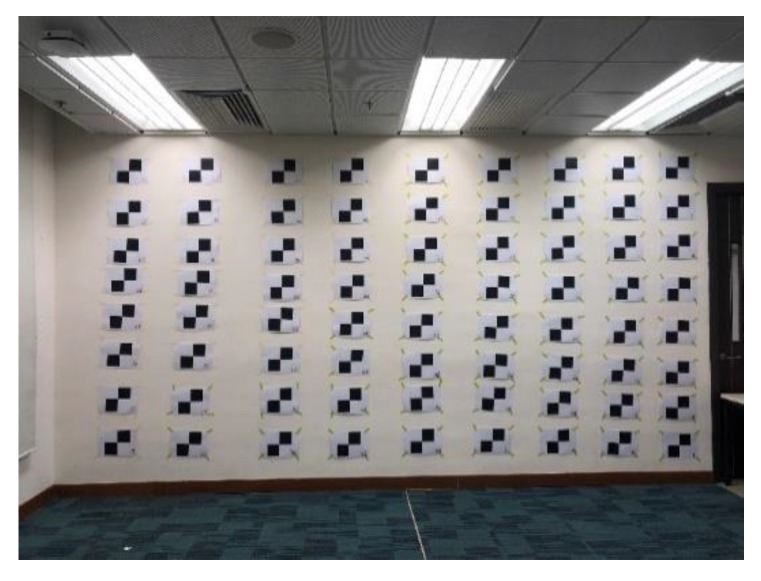
Test field for calibration.

In order to calibrate the SLP RGBD sensor, a calibration field is set in an open space. Figure 7 shows the test field used in this paper. The wall in the test filed is served as the control plane for calibration. The control plane is a plane with image identifiable markers and serves as the ground truth for calibration. The 3D coordinates of the image markers are measured by total station, an electronic surveying instrument that can measure high-precision 3D coordinates in mm level accuracy. Those image identifiable markers can provide a calibration dataset of image coordinates with its corresponding 3D coordinates in objective space. The calibration dataset is captured at a different distance to have a full coverage of the SLP RGBD sensor’s whole working range. In our test, the calibration dataset is captured from 0.5 m to 6.5 m with an interval about 0.5 m to cover the working range of the Structure Sensor. Since the wall with calibration mark covers less image area when the distance from the sensor to the wall increases, more than one station should be set for longer distance to make sure the calibration markers can cover the whole image. 

**Figure 8:**
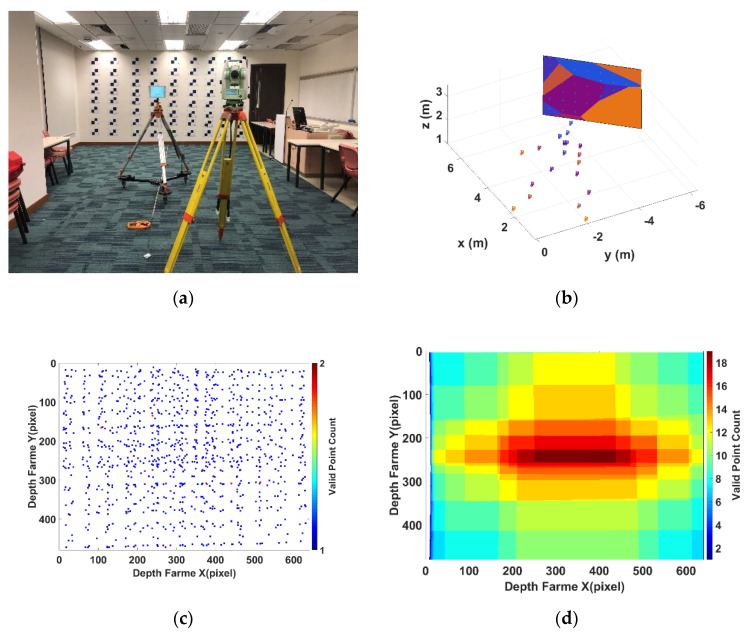
(**a**) Setup for data acquisition; (**b**) relationship between the stations and the fitted plane; (**c**) sparse valid data for estimation; (**d**) dense valid data calculated from plane fitting.

The data acquisition and processing procedure is as follows:

1. Establish a control plane by placing the control markers with known coordinates measured by total stations.
2. Fit the plane equation of the control plane.
3. Place the Structure Sensor in front of the control wall and take multiple observations (100 observations) at each designed distance (from 0.5 m to 6.5 m with an interval of about 0.5 m), as shown in Figure 8a,b.
4. Calculate the pixel-wise average depth frame for each distance to reduce the effect of random noise in each pixel.
5. Use the perspective-n-point algorithm [33] to calculate the camera extrinsic parameters for each distance based on the correspondences between the control wall and the depth frame.
6. Unify the coordinates system between Structure Sensor and the control wall based on the calculated camera extrinsic parameters in 5.
7. Calculate the ground truth depth for each distance based on the fitted control plane equation.
8. Calculate the ground truth disparity based on the processed ground truth depth.
9. Use the ground truth disparity to calculate the disparity error of the averaged disparity measurement in each distance.

The disparity error obtained after the data processing is used for model parameters estimation. The plane-fitted method is used to fit the equation of the control plane to provide adequate data for the parameter estimation. Figure 8b shows the different stations settled for data acquisition (camera markers) and the fitted planes based on different measurements (different colour on the control wall). The fitted plane can provide a significant amount of data for the estimation. If only the control markers measured in different distance are used for parameters estimation, the valid point that can be used for estimation is still too sparse to provide the full frame coverage, as shown in Figure 8c. By using the control markers, only 864 pixels within the frame have the associated disparity error and are valid for estimation, which covers about 0.28% of the whole 640 × 480 frame. By using the plane fitting to calculate the ground truth disparity for each control plane pixel, we can improve the number of valid pixel to 293.196, which cover about 94.44% of the whole frame. Furthermore, each pixel as shown in Figure 8d has multiple disparity errors that are measured from different distance. The comparison between Figure 8c,d shows a significant improvement of the valid data for calibration parameters’ estimation by the plane-fitting method. By minimizing the disparity errors for each pixel, we can further reduce random noise for a better parameter estimation.

### 3.2. Model Parameter Estimation

With the fitted plane ax+by+cz+d=0 and the camera pose $\left(R,t|R\in SO\left(3\right),\;t\in {\mathbb{R}}^{3}\right)$ are in the same reference system, the ground truth disparity error can be calculated through the following procedure. 

In the reference system of the fitted plane, the projection location $X_{i}\in {\mathbb{R}}^{3}$ of the depth pixel (u,v) on the fitted plane ax+by+cz+d=0 can be given by:

(15) $$X_{i}=\left(\frac{\left(P_{0}-L_{0}\right)\cdot \vec{n}}{\vec{l}\cdot \vec{n}}\right)\vec{l}+L_{0}$$

where $P_{0}$ can be any point on the fitted plane, $L_{0}$ is the coordinates of the camera centre t, $\vec{n}$ is the normal vector of fitted plane, and $\vec{l}$ is the line vector of the line pass through the camera centre t and depth pixel (u,v). 

Then the ground truth depth $Z_{i}$ for the pixel (u, v) in the depth frame can be calculated as:

(16) $$Z_{i}=\frac{\left|col_{3}\left(R\right)\cdot X_{i}-col_{3}\left(R\right)\cdot t\right|}{\sqrt{col_{3}\left(R\right)\cdot col_{3}\left(R\right)}}$$

where $col_{3}\left(R\right)$ stands for the third column of rotation matrix R. Based on Equation (3), the ground truth disparity $d_{i}^{\prime }$ can be given as:

(17) $$d_{i}^{\prime }=\frac{1-\alpha Z_{i}}{\beta Z_{i}}$$

Therefore, based on Equation (9), the disparity error $\delta_{d}$ of the depth pixel (u,v) can be obtained as:

(18) $$\delta_{d}=m\left(\delta_{c}^{ep}+\delta_{p}^{ep}\right)=d_{M}^{\prime }-d_{i}^{\prime }$$

**Figure 9:**
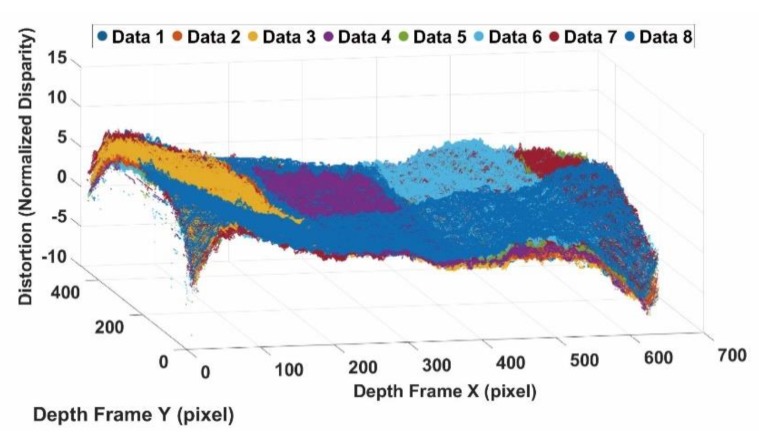
Observed error in normalized disparity.

The disparity errors obtained at different distances in the data acquisition are visualized in the following figure. Figure 9 shows the normalized disparity errors observed from 0.5 m to 6.5 m (with assorted colours). The different colours in Figure 9 represent the different measurements.

**Figure 10:**
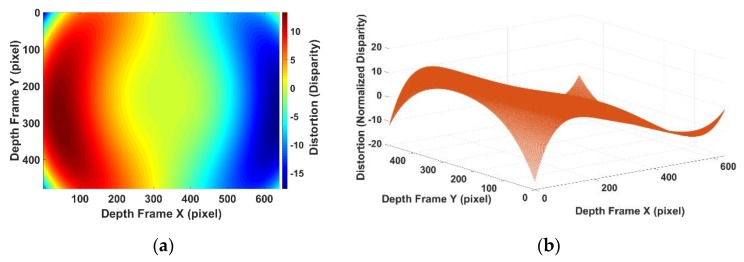
(**a**) Calibrated camera distortion in normalized disparity unit in image plane; (**b**) calibrated camera distortion in normalized disparity unit in 3D visualization.

Firstly, the IR camera distortion part can be calibrated separately by the standard checkboard calibration procedure [16]. It can be seen that the camera distortion is in both x axis and y axis of the image plane. While in disparity, only the x axis is required as the RGBD sensor only uses the pixel difference in the x axis to calculate the disparity. The distortion occurring in the y axis will not affect the disparity. Therefore, only the x axis is used as variables in the proposed disparity calibration model. Figure 10 shows the distortion model of the barrel distortion caused by camera lens that are calibrated by the standard camera calibration procedure. Figure 10a shows the modeled disparity correction for the whole frame coverage. The unit of x, the y axis of Figure 10a is the image pixel. The colour in Figure 10a indicates the disparity correction in normalized disparity. Figure 10b is a 3D visualization of Figure 10a. The x, y axis of Figure 10b is in the image pixel and z axis is in the normalized disparity. 

The parameters of Structure Sensor’s camera radial distortion and tangential distortion are estimated and listed in Table 1. The parameters are associated with the dimensionless normalized image coordinates, which are calculated from pixel coordinates by translating to the optical centre and dividing by the focal length in the pixel. Also, according to Equation (3), we can have $\beta =\frac{1}{fwm}$. Since β and the baseline w are known manufacturing parameters, and the focal length f can be obtained from standard camera calibration, the normalized coefficient m can be calculated.

**Figure 11:**
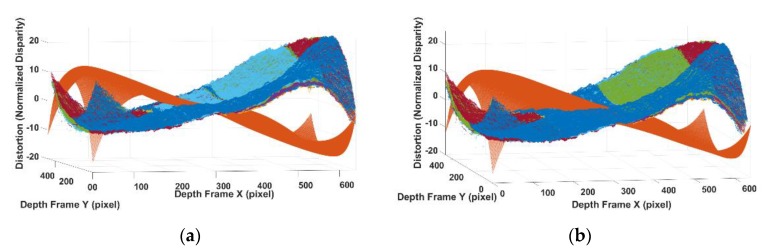
(**a**) Camera distortion and unaligned projector distortion; (**b**) camera distortion and aligned projector distortion.

After using the standard calibration method to model the camera-related barrel distortion, the pincushion distortion caused by projector can be simply separated by subtracting the observed data by the camera part (as shown in Figure 11a). As mentioned in Section 2, the remaining pincushion distortion is a centrosymmetric distortion with a shifted distortion centre based on the distance. How the projector caused the distortion overlay with the camera part is determined by the distance from the sensor to the object. As shown in Equation (8), the distortion centre of projector distortion can be calculated. The disparity errors caused by pincushion model shown in Figure 11a have different distortion centre based on the distance from the sensor to the object scene. Actually, the distortion parameters for these data captured at different distances are same. They have the same centrosymmetric shape but with different distortion centre in the image plane. By knowing the exact distance from sensor to the object scene, the shift caused by distance can be precisely calculated. All the disparity errors captured at different distances are then aligned back to the optical centre of the image frame for a better estimation of the distortion parameters, as shown in Figure 11b. 

**Figure 12:**
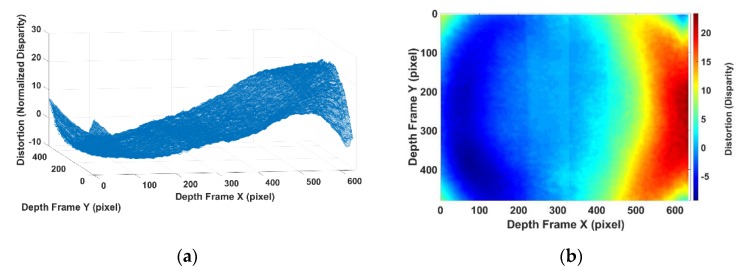
(**a**) Averaged processed projector distortion in 3D visualization; (**b**) averaged processed projector distortion in image plane.

By aligning the shifted projector distortion and taking the average disparity error in each pixel to reduce the effects of random noise, the processed data is regarded as the projector-caused disparity distortion. The aligned and averaged data shown in Figure 12 is the data processed to estimate the IR projector distortion parameters. The stripe pattern that appeared in Figure 12b is a normal phenomenal in SLP RGBD sensor [23,34]. The actual cause of this has not been revealed by the manufacture. Ref. [35] suggests the possible cause for this stripe pattern is the rolling shutter used in the IR camera. As the scale of the stripe pattern is much smaller than the distortion [6], the stripe pattern is uncalibrated in the proposed method. 

**Figure 13:**
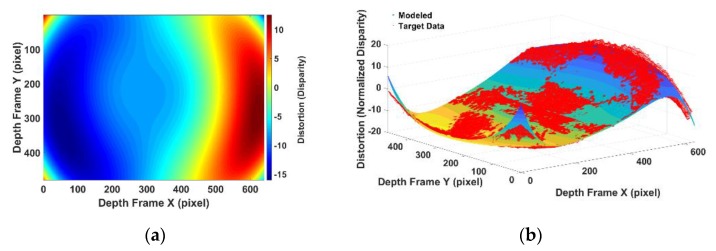
(**a**) Modelled projector distortion; (**b**) comparison of modelled projector error and actual data (red markers are the actual data; colourized surface is the modelled projector error).

Then, the Levenberg–Marquardt fitting algorithm [36] is used to minimized the cost function in Equation (10) and to estimate the parameters of the projector distortion model. The calibrated parameters are listed in Table 2: 

The parameter estimation result is shown in Figure 13a. The distortion model is compared with the actual observed distortion error. The comparison between the actual projector distortion and the modeled result (Figure 13b) shows that the calibration model can precisely match the actual disparity error. After calibrating the distortions caused by camera lens and projector lens, there are still some remaining errors of the observed disparity error. As shown in Figure 13b, there is still an increasing offset to the edge area between the actual disparity error and the proposed distortion model. 

**Figure 14:**
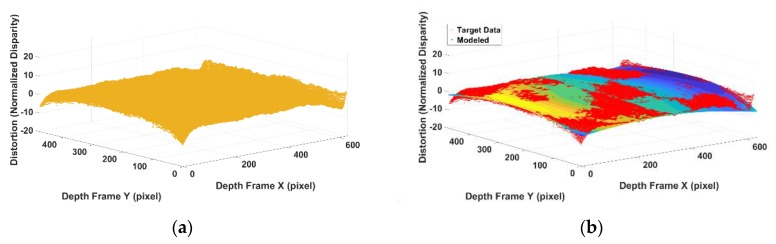
(**a**) Visualization of the remaining error after eliminate camera and projector distortions; (**b**) comparison of modelled IR cone error and actual error (red markers are the actual data, colourized surface is the modelled IR cone error).

The remaining part of the error according to [6,23] is caused by the reason that the images cannot be illuminated homogeneously, known as the infrared cone error. When the infrared light is emitted by the projector and cast on to the scene, the central area of the cone will have the highest illumination level. The illumination will decay towards the edge area. In the RGDB sensor, the illumination level peaks at the central area and decays in the edge area. As better illumination often benefits the pattern recognition performance [37], the offset caused by infrared cone in the central area is much smaller than the offset in the corner. As a result, it causes a systematic error across the depth frame with small offset in the central area and increasing offset towards the edge, as presented in Figure 14a. To model this systematic error caused by the infrared cone, a low order polynomial equation, shown in Equation (19), is applied to fit the offset caused by infrared cone. In Equation (19), *x, y* is the normalized image coordinates by optical centre in pixel, and $p_{00},p_{10},\ldots ,\;p_{03}$ are the polynomial parameters. The modeled result is shown in Figure 14b. The fitted parameters for Equation (19) are listed in Table 3.

(19) $$f\left(x,y\right)=p_{00}+p_{10}x+p_{01}y+p_{20}x^{2}+p_{11}xy+p_{02}y^{2}+p_{30}x^{3}+p_{21}x^{2}y+p_{12}xy^{2}+p_{03}y^{3}$$

**Figure 15:**
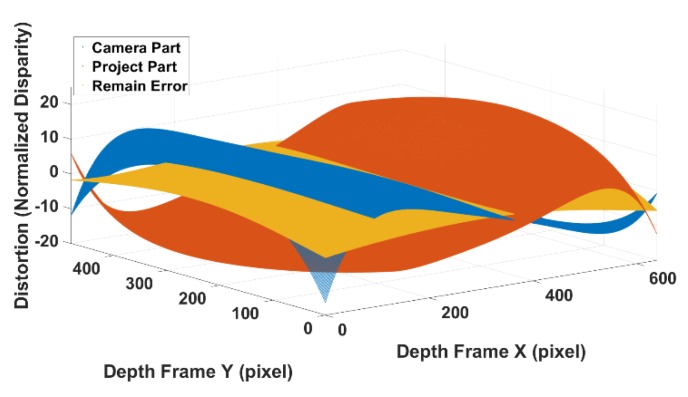
Three main parts of the distortion in the RGBD sensor.

To summarize, the calibration model consists of three parts (Figure 15): (1) a barrel distortion model calibrated with classic checkboard camera calibration, which is fixed in the optical centre; (2) a pincushion distortion model based on the proposed procedure, of which the effects on the disparity is a fixed centrosymmetric shape while this has a varying distortion centre on the image plane; (3) a low order polynomial equation for the error caused by infrared cone, which has the same varying centre as the pincushion distortion because the cone is caused by the projector when it illuminates the scene. Combining these three parts, we can achieve a range-independent disparity-based calibration model for SLP RGBD sensors. Since the calibration model is developed based on the actual physical model of the SLP RGBD sensor’s distortion, the proposed method is a range independent model. No range-based parameter selection is required in the proposed method. It can provide the full range coverage with one single model of 20 parameters (5 parameters for barrel distortion, 5 parameters for pincushion distortion, and 10 parameters for the infrared cone related error). Considering the overlapping mechanism between the barrel distortion and pincushion, the proposed method can use a non-centrosymmetric distortion model to provide full frame calibration of the depth frame. The offset in the edge area is largely calibrated. By accurately calibrating the edge area, the proposed method can significantly increase the quality of the depth frame and provide additional information that is missed out due to the large error.

## 4. Calibration Model Performance

Two experiments were designed to test the performance of the proposed calibration method with the SLP RGDB sensor, Structure Sensor. The performance of the calibration model was evaluated by comparing the difference between the modeled error and the actual error. Then a more straightforward showcase of the calibrated point cloud and the uncalibrated point cloud was provided to demonstrate the performance of the proposed calibration model. 

### 4.1. Model Evaluation

**Figure 16:**
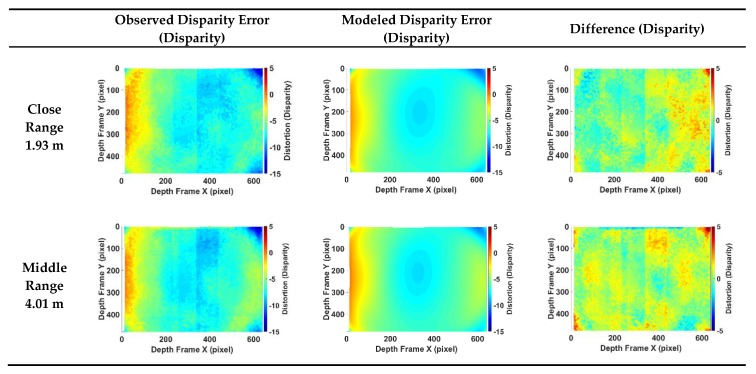

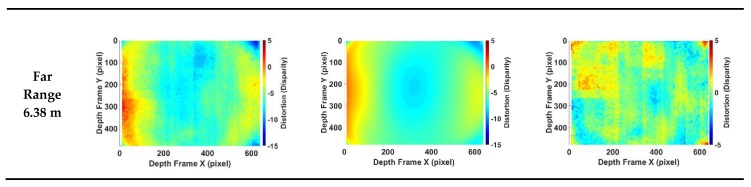
Comparison of ground truth of disparity error, the modelled disparity error based on the proposed model, and the difference between the ground truth and the modelled data.

To test the calibration method’s performance, a test dataset that followed the same setup as mentioned in Section 3.1 was obtained in an unfamiliar environment. The proposed calibration model was put into a test to compare the difference between the actual disparity error and the modelled error by the proposed method. The test dataset was captured from 2 m to 7 m with an interval of about 0.5 m. The example shown in Figure 16 is three typical results for close range (1.93 m), middle range (4.01 m) and far range (6.38 m). The first column is the actual observed disparity error. The second column is the modeled disparity error based on the proposed calibration model. The last column is the difference between the first two columns. For all the figures presented in Figure 16, the x and y axis are in image pixels and the colour indicates the value of the disparity error at that pixel location in the normalized disparity. As shown in Figure 16, the proposed model can accurately model the actual error. The difference between the modelled error and the actual error is minor. The root mean squared error (RMSE) of the overall modelled error is 0.76 disparity units. While two right corners still have some large offset that cannot be accurately modelled. It may be caused by the less enough data in the corner area.

### 4.2. Point Cloud Correction

**Figure 17:**
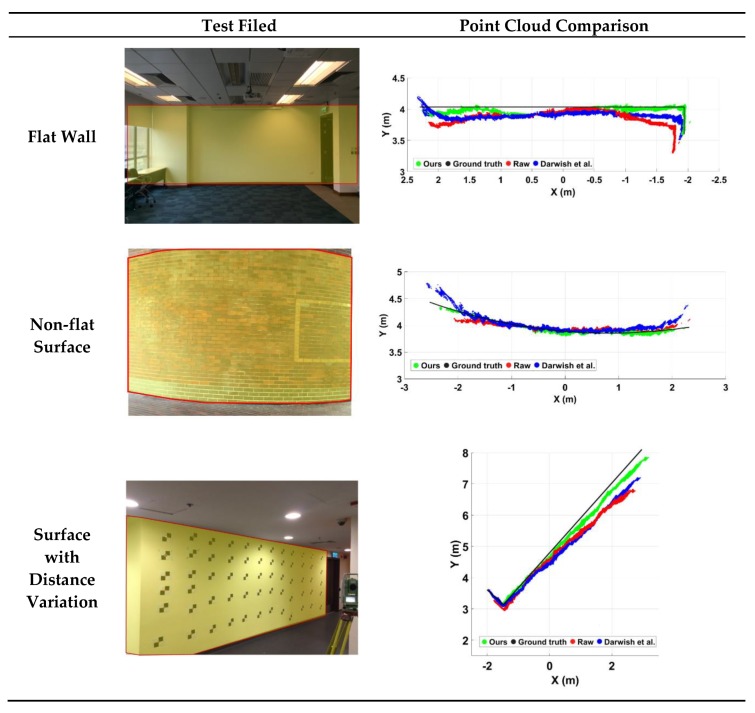
Calibration comparison on flat surface, non-flat surface, and surface with distance variation.

A more direct method to evaluate the quality of the calibration model is the visualization of data quality improvement of the measured point cloud. The proposed method was tested in three different scenes: (1) flat surface; (2) non-flat (curved) surface; and (3) flat surface with distance variation, shown in Figure 17. Darwish et al.’s calibration method [19] was selected as the reference of existing calibration performance for the SLP RGBD sensor. As Figure 17 shows, the proposed calibration model improves the point cloud accuracy in all three scenes. The validations in non-flat surface and the surface with depth variation indicate the calibration model was applicable in all ranges. The 3D data between the sampling distances can also be calibrated by the proposed method.

**Figure 18:**
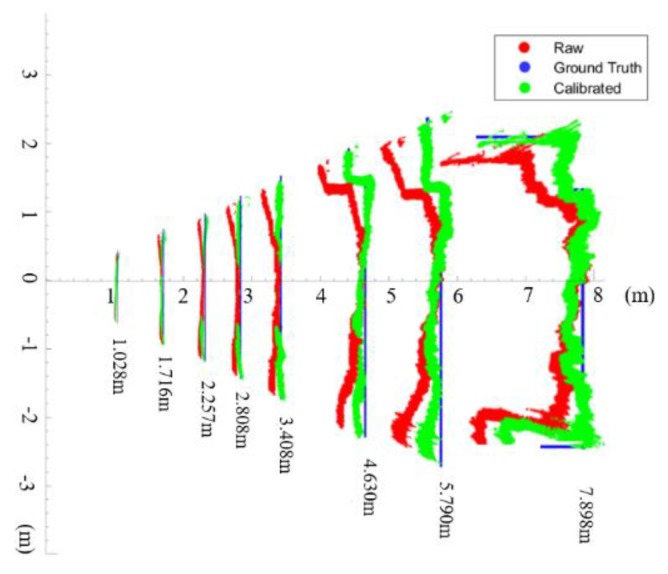
Full range result of the calibration model.

Figure 18 shows the full range improvements of the calibration model. For the full range of the SLP RGBD sensor’s working range, the proposed method can greatly improve the quality of the measured point cloud. The improvement of the edge area shown in Figure 18 indicates the full frame coverage of the proposed calibration method. The calibration model is derived from the actual physical properties of how the camera distortion and projector distortion combined in the image frame. Therefore, the proposed calibration can accurately model the actual error to greatly improve the point cloud quality of the low-cost sensor, especially in the far range and edge area of the depth frame. 

**Figure 19:**
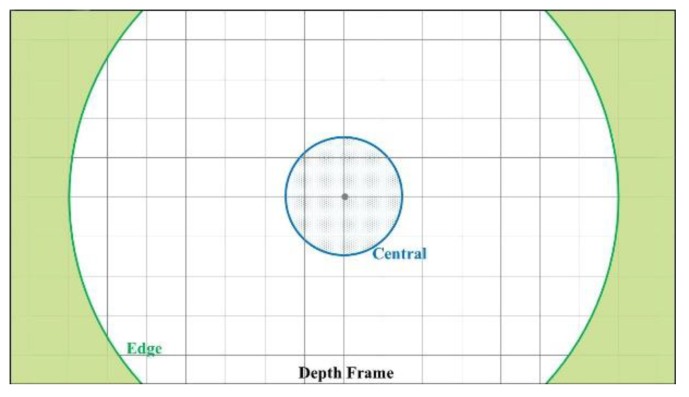
Central region and edge region of depth frame.

The accuracy improvement of the proposed calibration method can be demonstrated from two different regions within the depth frame. As shown in Figure 18, the offset of the depth measurement increases differently between the central region and edge region of the depth frame. The central region that is near the optical centre of the camera suffers much less errors compared to the edge region. The increasing pace of the offset in the edge region is much faster than the central part. Therefore, it is reasonable to demonstrate the accuracy improvements for different parts of the frame separately. Figure 19 shows how the central region and edge region are defined in the depth frame. A central subset and an edge subset of depth measurement are selected based on Figure 19 for each tested distance. The mean value of each subset is used for accuracy evaluation. 

**Figure 20:**
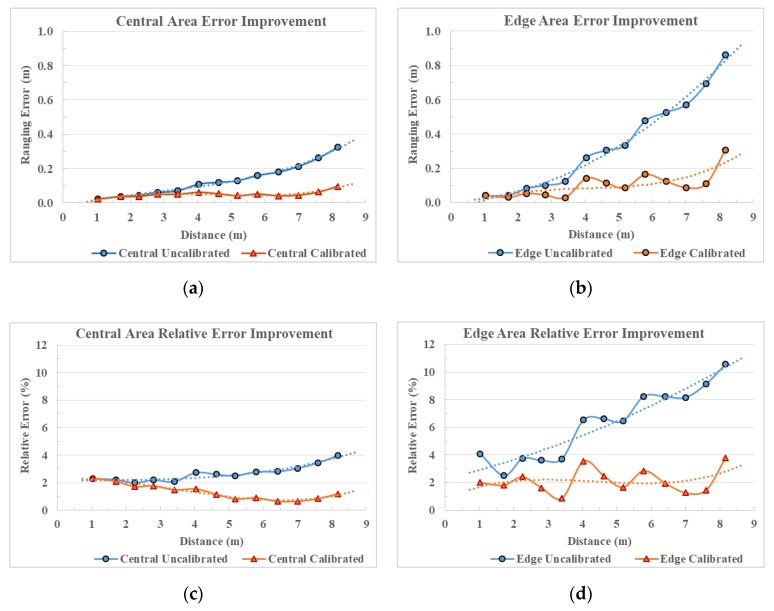
Accuracy and relative error ration to distance for central and edge area with trend included. (**a**) Ranging error improvement in central area; (**b**) ranging error improvement in edge area; (**c**) relative error improvement in central area; and (**d**) relative error improvement in edge area.

Figure 20 shows the accuracy improvement of the proposed method. The x axis in all figures is the ground truth distance in meters. The y axis in Figure 20a,b is the ranging error in meters. The y axis in Figure 20c,d is the relative ranging error to ground truth ratio in percentages. The markers linked with lines in the figures are the original observed data at different distances. The dot lines are the polynomial fitted trend for its corresponding data with the same colour. Figure 20a,b show the accuracy improvement of applying a calibration model for both central area and edge area of the depth frame. As shown in Figure 20a, for the central region, the uncalibrated range normally has around 30 cm ranging error at 8 m distance. After calibration, the central region will only have about 10 cm error at 8 m distance. The accuracy is improved by about 70% in the central region. The improvement for the edge area is more significant as the proposed model is developed from the actual mechanism of the distortion and provides the full frame coverage. As shown in Figure 20b, the uncalibrated edge region suffers a large error (86 cm) at 8 m distance. After calibration, the edge region calibrated depth reduces the error form 86 cm to 30 cm at 8 m distance, which is improved by about 65%. As for the relative error ratio (error to distance) that is shown in Figure 20c, the relative error of the central area is largely reduced through the entire working range from an overall 4% of the distance to about 1% of the distance. The improvement for the edge area shown in Figure 20d is more significant as the proposed model is developed from the actual mechanism of the distortion and provides the full frame coverage. The edge region has a large 11% relative error of the ranging distance before the calibration. It suffers a much more severe distortion compared to the central region’s 4% relative error at the same distance. With the calibration model, the relative error in the edge region can be reduced from 10% of the distance to 3% of the distance. It is also indicated in Figure 20d that before calibration the relative error/distance ratio increases with the distance. After calibration, the calibration data can provide stable 2% to 3% ranging data in the edge region and 1% to 2% ranging data in the central region for the full range. 

## 5. Conclusions and Future Work

The RGBD sensor has a promising future to replace the high-cost 3D laser scanner and be applied in high-precision applications such as robotics, high-precision localization and mapping. The radial systematic error in the depth frame significantly limits its potential applications. Targeting this problem, this paper presented range-independent disparity-based calibration method for the SLP RGDB sensor. By revealing the real cause and forming mechanism of the non-centrosymmetric depth distortion, the proposed calibration method targets the disparity unit rather than the depth. By applying the calibration model in disparity and calculating the calibrated depth based on the calibrated disparity, the calibration model is independent of distance. No additional distance-based calibration or parameters look up table is required for the proposed method. With only one model with 20 parameters, the proposed calibration method can provide a full range coverage for the SLP RGBD sensor. A new non-centrosymmetric distortion calibration model for the normalized disparity is proposed in this paper based on the discussion on the form of the mechanism of the SLP RGBD sensor distortion. The proposed non-centrosymmetric distortion model can significantly reduce the large offset in the edge area of the depth frame. Since the heavily distorted edge area now can be calibrated to a similar accuracy level to the central area, more usable and valid information can be extracted and used to benefit applications such as SLAM, robotic exploration and obstacle avoidance. A new two-step calibration procedure is also developed in this paper to calibrate the barrel distortion caused by the IR camera lens, pincushion caused by the IR projector lens, and the systematic error caused by the IR cone. 

In the experimental results, the full frame and full-range coverage of the proposed calibration method is demonstrated. The comparison between the calibrated and uncalibrated point cloud clearly shows that the systematic errors in the measured point cloud have been removed by the calibration model. The significant offset in the edge area of long-range depth is reduced by the proposed model from 86 cm to 30 cm, which means that the relative error is reduced from 11% to 3% of the range distance. Overall, at far range the proposed calibration method can improve the depth accuracy by 70% in the central region of depth frame and 65% in the edge region.

Further work will study the long-term stability of calibration parameters for the consumer-grade RGBD sensor. Other potential systematic error sources, such as illumination condition, temperature, humidity, and air refractive index, will be investigated for a more comprehensive calibration model. 

## Figures and Tables

**Table 1:** Calibrated distortion parameters of infrared (IR) camera.

Camera Parameter	Radial Distortion			Tangential Distortion		Normalized Coefficient
	$K_{1}^{c}$	$K_{2}^{c}$	$K_{3}^{c}$	$P_{1}^{c}$	$P_{2}^{c}$	m
Structure Sensor	−0.0578	0.1248	1.0212 × 10^−4^	−0.0010	−6.0139 × 10^−4^	7.9418

**Table 2:** Calibrated distortion parameters of IR projector.

Projector Parameter	Radial Distortion			Tangential Distortion	
	$K_{1}^{p}$	$K_{2}^{p}$	$K_{3}^{p}$	$P_{1}^{p}$	$P_{2}^{p}$
Structure Sensor	0.0474	−0.0714	−0.1014	0.0019	1.4390 × 10^−4^

**Table 3:** Fitted polynomial parameters for infrared cone error.

Polynomial Parameters	Value	Polynomial Parameters	Value
$p_{00}$	0.5432	$p_{02}$	−0.6831
$p_{10}$	−0.0579	$p_{30}$	0.0604
$p_{01}$	0.1775	$p_{21}$	0.0535
$p_{20}$	0.1471	$p_{12}$	−0.0368
$p_{11}$	0.0113	$p_{03}$	−0.0515
